# Histone 3.3 hotspot mutations in conventional osteosarcomas: a comprehensive clinical and molecular characterization of six H3F3A mutated cases

**DOI:** 10.1186/s13569-017-0075-5

**Published:** 2017-05-04

**Authors:** Christian Koelsche, Daniel Schrimpf, Lars Tharun, Eva Roth, Dominik Sturm, David T. W. Jones, Eva-Kristin Renker, Martin Sill, Annika Baude, Felix Sahm, David Capper, Melanie Bewerunge-Hudler, Wolfgang Hartmann, Andreas E. Kulozik, Iver Petersen, Uta Flucke, Hendrik W. B. Schreuder, Reinhard Büttner, Marc-André Weber, Peter Schirmacher, Christoph Plass, Stefan M. Pfister, Andreas von Deimling, Gunhild Mechtersheimer

**Affiliations:** 1 Department of Neuropathology, Institute of Pathology, University Hospital Heidelberg, Im Neuenheimer Feld 224, 69120 Heidelberg, Germany; 2 German Cancer Consortium (DKTK), CCU Neuropathology, German Cancer Research Center (DKFZ), Heidelberg, Germany; 3 Institute of Pathology, University Hospital Cologne, Cologne, Germany; 4 Department of Pediatric Oncology, Hematology and Immunology, University Hospital Heidelberg, Heidelberg, Germany; 5 Division of Pediatric Neurooncology, German Cancer Consortium (DKTK), German Cancer Research Center (DKFZ), Heidelberg, Germany; 6 Department of Orthopedics and Traumatology, University Hospital Heidelberg, Heidelberg, Germany; 7 Division of Biostatistics, German Cancer Research Center (DKFZ), Heidelberg, Germany; 8 Division of Epigenomics and Cancer Risk Factors, German Cancer Research Center (DKFZ), Heidelberg, Germany; 9 Genomics and Proteomics Core Facility, Microarray Unit, German Cancer Research Center (DKFZ), Heidelberg, Germany; 10 Gerhard Domagk Institute of Pathology, University Hospital, Muenster, Germany; 11 Institute of Pathology, University Hospital, Jena, Germany; 12 Department of Pathology, Radboud University Hospital, Nijmegen, The Netherlands; 13 Department of Orthopedics, Radboud University Hospital, Nijmegen, The Netherlands; 14 Clinic of Diagnostic and Interventional Radiology, University Hospital Heidelberg, Heidelberg, Germany; 15 Department of General Pathology, Institute of Pathology, University Hospital Heidelberg, Im Neuenheimer Feld 224, 69120 Heidelberg, Germany

**Keywords:** Mutation, H3.3, H3F3A, HIST1H2BB, KLLN, PTEN, Osteosarcoma, Giant cell tumor of bone, DNA methylation, Copy number alteration

## Abstract

**Background:**

Histone 3.3 (H3.3) hotspot mutations in bone tumors occur in the vast majority of giant cell tumors of bone (GCTBs; 96%), chondroblastomas (95%) and in a few cases of osteosarcomas. However, clinical presentation, histopathological features, and additional molecular characteristics of H3.3 mutant osteosarcomas are largely unknown.

**Methods:**

In this multicentre, retrospective study, a total of 106 conventional high-grade osteosarcomas, across all age groups were re-examined for hotspot mutations in the H3.3 coding genes *H3F3A* and *H3F3B*. H3.3 mutant osteosarcomas were re-evaluated in a multidisciplinary manner and analyzed for genome-wide DNA-methylation patterns and DNA copy number aberrations alongside H3.3 wild-type osteosarcomas and *H3F3A* G34W/L mutant GCTBs.

**Results:**

Six osteosarcomas (6/106) carried *H3F3A* hotspot mutations. No mutations were found in *H3F3B*. All patients with *H3F3A* mutant osteosarcoma were older than 30 years with a median age of 65 years. Copy number aberrations that are commonly encountered in high-grade osteosarcomas also occurred in *H3F3A* mutant osteosarcomas. Unlike a single osteosarcoma with a *H3F3A* K27M mutation, the DNA methylation profiles of *H3F3A* G34W/R mutant osteosarcomas were clearly different from H3.3 wild-type osteosarcomas, but more closely related to GCTBs. The most differentially methylated promoters between *H3F3A* G34W/R mutant and H3.3 wild-type osteosarcomas were in *KLLN/PTEN* (p < 0.00005) and *HIST1H2BB* (p < 0.0005).

**Conclusions:**

H3.3 mutations in osteosarcomas may occur in *H3F3A* at mutational hotspots. They are overall rare, but become more frequent in osteosarcoma patients older than 30 years. Osteosarcomas carrying *H3F3A* G34W/R mutations are associated with epigenetic dysregulation of *KLLN/PTEN* and *HIST1H2BB*.

**Electronic supplementary material:**

The online version of this article (doi:10.1186/s13569-017-0075-5) contains supplementary material, which is available to authorized users.

## Background

Osteosarcoma is a malignant bone forming neoplasm with a broad spectrum of morphologies [1]. Conventional high-grade osteosarcoma is the most common subtype and typically arises in the long bones of the extremities with a predilection to the metaphysis, although any bone of the skeleton may be affected. The incidence of osteosarcomas has a bimodal age distribution with a first peak in the second decade of life and a second smaller peak in older adults [1, 2].

The etiology of osteosarcomas is still largely unknown. However, several genetic syndromes (e.g. Li-Fraumeni and hereditary retinoblastoma) greatly increase the risk of developing osteosarcomas, especially in younger patients [3, 4]. Osteosarcomas in older patients often develop from a precursor bone lesion that is prone for malignant transformation (i.e. Paget disease) or may occur after radiation treatment for other diseases, typically after a latency period of several years [1, 5, 6].

The genomic landscape of osteosarcomas is complex [7]. A remarkable attribute of primary and secondary conventional osteosarcomas is their chromosomal instability leading to high aneuploidy [8]. Chromosomal regions frequently affected by somatic structural variations and copy number alterations often contain cell-cycle regulating genes such as *TP53*, *RB1*, or c-*MYC* [9, 10]. Sequencing studies revealed recurrent mutations affecting these genes, supporting their major role in osteosarcoma development [8]. However, the mutational make-up of osteosarcomas is versatile, as some osteosarcomas also present with molecular signatures reminiscent of BRCA1/2 deficient tumors [10]. *Chromothripsis*, a single cellular catastrophic event resulting in extensive DNA rearrangements and DNA copy number changes, has been observed in roughly one-third of osteosarcomas [11].

Alternative lengthening of telomeres (ALT), a telomere maintaining mechanism compensating progressive telomere attrition by homologous recombination of telomere sequences, is associated with chromosomal instability in osteosarcomas [12]. Almost all osteosarcomas utilize ALT, highlighting its particular importance in these tumors [8, 13]. Somatic mutations in the H3.3-ATRX-DAXX chromatin-remodeling pathway have been linked to ALT in different tumor entities [14, 15]. *ATRX* (α-thalassaemia/mental retardation syndrome X-linked) mutations, which result in a loss of its nuclear expression, were found in osteosarcomas at a frequency of 20–30% [8, 16]. Loss of DAXX (death-domain associated protein) expression has not yet been observed in any sarcoma [17]. However, a high frequency of mutations in the two genes encoding histone 3 variant 3 (H3.3) was revealed in certain bone tumors. Giant cell tumors of bone (GCTBs) almost always carry mutations in *H3F3A* at codon 34, whereas chondroblastomas are characterized by a high frequency of *H3F3B* mutations at codon 36 [18–20]. Little is known about H3.3 mutations in osteosarcomas. Previous studies described single high-grade osteosarcomas carrying H3.3 mutations, whereas other series did not. So the question arises whether osteosarcomas carrying H3.3 mutations really exist or might rather be malignant variants of GCTBs [8, 18–21].

The present study addresses these issues by determining the frequency of H3.3 mutations across the entire age spectrum in de novo high-grade osteosarcomas of the conventional type. Genome-wide DNA methylation and DNA copy number profiling was applied to gain further molecular insight into osteosarcomas carrying H3.3 mutations.

## Methods

### Tissues specimens

Tissue samples of 106 de novo high-grade osteosarcomas of the conventional type (86 primary tumors, 3 recurrences, 17 metastases) and 14 GCTBs, one of them with signs of malignancy in the local relapse two years after surgical treatment, serving as H3.3 G34 mutant bone tumor control group were collected from the Institutes of Pathology of the University Hospital Heidelberg, Cologne, Jena (Germany), and Nijemegen (the Netherlands). Patients’ characteristics are outlined in Additional file 1: Table S1. Tissue samples were banked in the archives of the Department of Pathology and administered by the tissue bank of the National Center for Tumor Diseases (NCT), Heidelberg. Initial diagnoses were established by interdisciplinary oncology boards and were based on radiological findings, standard histopathological criteria in conjunction with immunohistochemical and molecular analyses according to the current WHO classification [1]. H3.3 mutant osteosarcomas were correlated with clinical information and were histologically and radiologically re-evaluated where available. The local ethics committee approved these analyses.

### DNA extraction and Sanger sequencing of *H3F3A/B* mutational hotspots

Source material for DNA extraction was formalin-fixed and paraffin-embedded (FFPE) tumor tissue. Representative tumor tissue with highest available tumor content was chosen for genomic DNA isolation, as microscopically documented on H&E stained paraffin sections. In GCTBs, which are known for their prominent non-tumoral giant cell component, areas with predominant tumor cells (at least 70%) were selected for DNA extraction. The Maxwell^®^ 16FFPE Plus LEV DNA Kit was applied on the automated Maxwell device (Promega, Madison, WI, USA) according to the manufacturer’s instructions. Polymerase chain reaction was performed with 20 ng of DNA under conditions as previously described [19]. Two microliters of the amplification product were submitted to bidirectional sequencing using the BigDye Terminator v3.1 Sequencing Kit (Applied Biosystems, Foster City, CA, USA). Sequences were determined using an ABI 3500 Genetic Analyzer (Applied Biosystems) and the Sequence Pilot version 3.1 (JSI-Medisys, Kippenheim, Germany) software.

### Genome-wide DNA methylation data generation and pre-processing

The Illumina Infinium HumanMethylation450 (450 k) array was used to assess the DNA methylation status of 482,421 CpG sites (Illumina, San Diego, USA), according to the manufacturer’s instructions at the Genomics and Proteomics Core Facility of the German Cancer Research Center (DKFZ) Heidelberg. DNA methylation data were normalized by performing background correction and dye bias correction (shifting of negative control probe mean intensity to zero and scaling of normalization control probe mean intensity to 10,000, respectively). Probes targeting sex chromosomes, probes containing multiple single nucleotide polymorphism and those that could not be uniquely mapped were removed. In total, 438,370 probes were kept for analysis.

### Unsupervised clustering, copy number profiling and identification of differentially methylated regions

For unsupervised hierarchical clustering, we selected 20,000 probes that showed the highest standard deviation (SD) of beta values across the entire dataset. Samples were hierarchically clustered using the 1-Pearson correlation coefficient as distance measure and average linkage. DNA methylation probes were reordered using Euclidian distance and complete linkage.

Copy number profiles were generated from the 450 k raw data using the ‘conumee’ R package and assessed manually. The dmpFinder method of the R package minfi was used for the identification of differentially methylated regions [22]. Both packages are available from the Bioconductor repository (http://www.bioconductor.org).

### Statistical analysis

A two-sided t test was used to test for differences between the mean values for continuous variables. P values of less than 0.05 were considered significant.

## Results

### *H3F3A* mutations occur in high-grade osteosarcomas of elderly patients

**Fig. 1:**
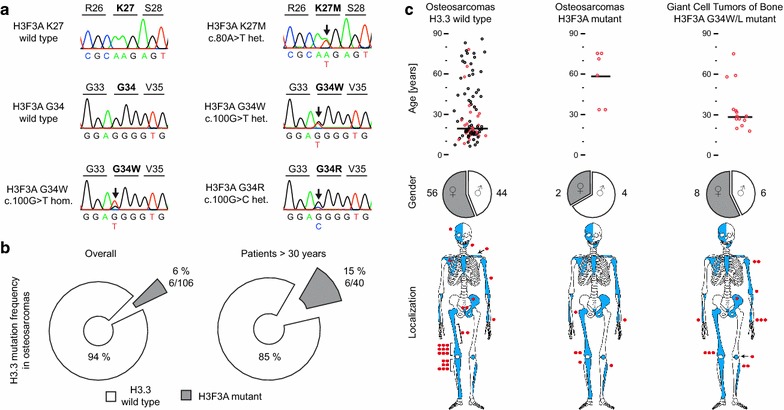
*H3F3A* mutations and patients characteristics of the study group. **a** Chromatograms showing the *H3F3A* sequence spanning codon 27 and 34 illustrate the wild-type status and mutations that exchange either the amino acid lysine with methionine (K27M), glycin (G34) with tryptophan (G34W) or with arginine (G34R) **b** *Charts* show the overall incidence of *H3G3A* G34W mutations in our series of osteosarcomas over all age groups and in the group of patients older than 30 years. **c** Osteosarcomas are separated in wild-type and mutant regarding their *H3F3A* status. Giant cell tumors of bone act as control. The age distribution is demonstrated for each case. The *black bar* indicates the median age. Gender distribution is illustrated in the form of a diagram. The site of occurrence is only shown for cases that were further analysed for methylome and copy number profiling. Each *ring*/*dot* represents a case. *Red color codes* for cases that were further analyzed for methylome and copy number profiling

Targeted sequencing of the two genes encoding histone 3 variant 3 (*H3F3A* and *H3F3B*) in 106 de novo high-grade osteosarcomas revealed six *H3F3A* mutant cases (Fig. 1a). One case carried a c.80A>T heterozygous mutation leading to an amino acid exchange of lysine by methionine at codon 27 (K27M). Another osteosarcoma presented with a c.100G>C heterozygous mutation leading to an exchange of glycin (G) by arginine (R) at codon 34. Four cases carried a c.100G>T mutation, one hemizygous and three heterozygous, leading to an exchange of glycin (G) by tryptophan (W). No mutation was detected in *H3F3B*. Patients with *H3F3A* mutant osteosarcomas were diagnosed at higher age (median age 65 years; range 34–75 years vs median age 18 years; range 2–86 years) and were significantly older (p < 0.05) compared to the control group of *H3F3A* mutant GCTBs (median age 29 years, range 18–75 years). Hence, the incidence increased from overall 5% (6/106) to 15% (6/40) in osteosarcoma patients over 30 years of age (Fig. 1b, c). The six *H3F3A* mutant osteosarcomas affected the long bones and were distributed similar to H3.3 wild-type osteosarcomas and GCTBs. They occurred in the proximal and distal femur, proximal tibia, proximal fibula, or the distal radius (Fig. 1c; Table 1).

**Table 1:** Clinical characteristics of the six H3.3 mutant osteosarcomas

#	H3.3 status	Age at Dx (years)	Gender	Tumor location	Histology	Therapy protocol	Follow-up (months)	Status	Comments
77896	H3F3A G34W	59	Male	Distal radius	Osteoblastic	According to EUROBOSS	52	Progressive disease	Local recurrence, lung, pleura and liver metastases 49 months after Dx
79428	H3F3A G34W	75	Male	Proximal tibia	Osteoblastic/fibroblastic	Unknown	–	Unknown	No further clinical documentation available
84676	H3F3A G34W	71	Male	Distal femur	Osteoblastic/fibroblastic	According to EUROBOSS	–	Unknown	No further clinical documentation available
84712	H3F3A G34W	34	Female	Proximal fibula	Osteoblastic	According to euramos	17	Complete remission	Initially dx of an aneurysmal bone cyst treated by excochleation with bone grafting, continuous progress, re-biopsy 1 year later with dx osteosarcoma
94316	H3F3A G34R	75	Male	Distal femur	Osteoblastic/fibroblastic	No further treatment	28	Death of disease	Local recurrence and lung metastases 19 months after Dx
94314	H3F3A K27M	34	Female	Proximal femur	Osteoblastic/fibroblastic	According to EURAMOS	27	Death of disease	Traumatic fracture of the proximal femur treated with nail osteosynthesis 7 years before Dx, bone and lung metastases, bone infection

### Radiological features and histological patterns are indistinguishable between *H3F3A* mutant and H3.3 wild-type osteosarcomas

**Fig. 2:**
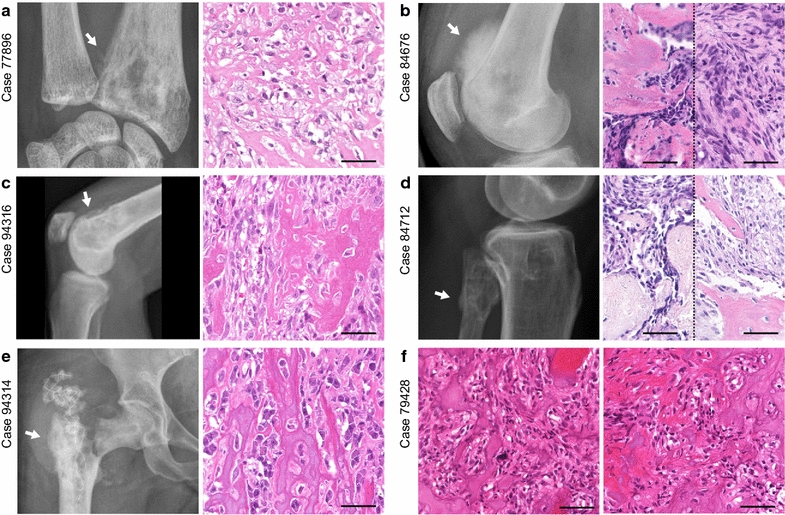
Radiologic appearance and morphological phenotypes of *H3F3A* mutant osteosarcomas. **a** Anteroposterior radiograph of the left wrist shows a lesion in the epi- and metaphysis of the distal radius (*left*). Histology revealed an osteoblastic osteosarcoma with pronounced nuclear atypia, brisk mitotic activity and a compact, extensive sclerosing osteoid matrix. **b** Lateral radiograph of the right knee shows a lesion in the distal femoral metaphysis with infiltration of the epiphysis and prominent extension into the soft tissue. The tumor was composed of pleomorphic, relatively plump and focally spindle-shaped tumor cells depositing osteoid in plaques (mixed osteoblastic/fibroblastic osteosarcoma). **c** Lateral radiograph of the right knee shows a lesion in the distal femoral meta- and epiphysis with subchondral extension and infiltration of the joint. The tumor (mixed osteoblastic/fibroblastic osteosarcoma) was predominantly composed of plump tumor cells producing coarse plaque-like osteoid. **d** Lateral radiograph of the right knee shows a lesion in the proximal fibula (caput fibulae). Histology revealed an osteoblastic osteosarcoma with highly pleomorphic tumor cells focally producing coarse and lace-like osteoid deposits. **e** Anteroposterior radiograph of the hip joint shows a lesion in the intertrochanter and subtrochanter region of the proximal femur. Histology revealed a mixed osteoblastic/fibroblastic osteosarcoma with coarse osteoid production. **f** X-ray imaging of the proximal tibia was not available in case 79,428. Histologically this bone lesion presented as osteosarcoma (mixed osteoblastic/fibroblastic subtype) with neoplastic bone production that was focally arranged in a coarse lace-like pattern. Foci with necrosis and dystrophic calcification were noted. *White arrows* indicate the corresponding lesion (**a**–**e**). *Scale bars* equal 50 µm (**a**–**f**)

Radiological data was available for re-evaluation in five of six *H3F3A* mutant osteosarcomas (Fig. 2; Additional file 2: Figure S1). At radiographic examination, the tumors to a variable extent demonstrated a mixed pattern of sclerotic opacities with mineralization and lytic areas violating the cortex. The infiltrative growth pattern was associated with expansive growth into the surrounding soft tissue. Some cases presented with prominent soft-tissue masses (e.g. Fig. 2b). On MR imaging, where available, these masses partly wrapped around the circumference of the affected bone (Additional file 2: Figure S1).

All six *H3F3A* mutant cases showed histological features indistinguishable from other conventional high-grade osteosarcomas (Fig. 2; Additional file 3: Figure S2). Mitotic activity was variable with presence of atypical mitotic figures. Neoplastic bone formation was observed in all *H3F3A* mutant osteosarcoma, although to a variable extent. Of note, giant cells were sparse to absent in all six *H3F3A* mutant osteosarcomas.

### DNA methylation profiling separates *H3F3A* G34 mutant osteosarcomas from H3.3 wild-type counterparts

**Fig. 3:**
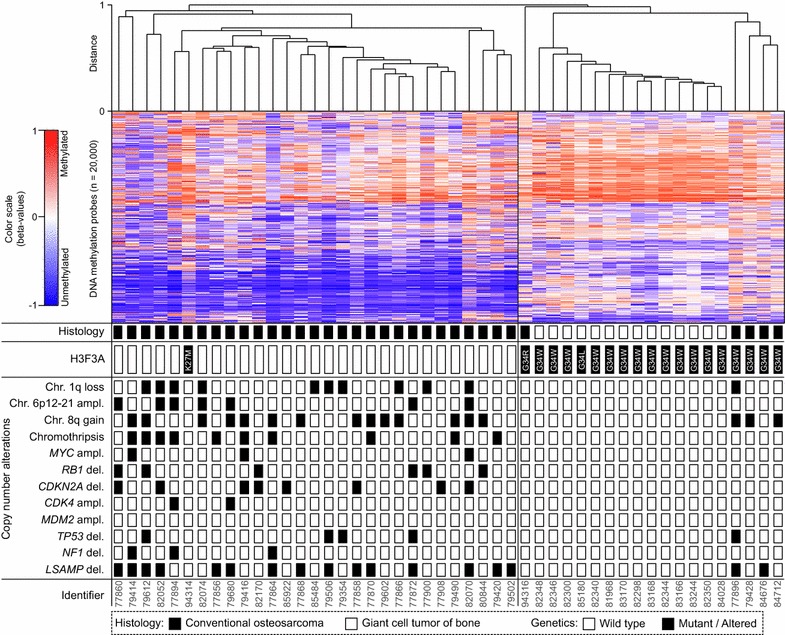
Methylome and copy number profiling. The heatmap depicts unsupervised hierarchical clustering of methylation levels of the top 20,000 most variant probes (highest standard deviation). Each* row* represents a probe and each* column* a sample. The level of DNA methylation (beta value) is represented with a *colour scale* as depicted. Fourteen giant cell tumors of bone carrying H3F3A G34 mutations served as control group. For each sample (n  =  48), histological diagnosis, mutational status of H3F3A and selected chromosomal copy number alterations are indicated

We next investigated the six *H3F3A* mutant and 28 H3.3 wild-type osteosarcomas and 14 *H3F3A* G34W/L mutant GCTBs for genome-wide DNA methylation patterns applying the 450 k DNA methylation array (Fig. 3). Unsupervised hierarchical clustering identified two distinct DNA methylation clusters (Fig. 3, upper panel). The first cluster was composed of H3.3 wild-type osteosarcomas and the single osteosarcoma carrying a *H3F3A* K27M mutation. This particular case was indistinguishable from that of H3.3 wild-type osteosarcomas. The second methylation cluster was composed of the five *H3F3A* G34 mutant osteosarcomas and the *H3F3A* G34 mutant GCTB control cases. Within this cluster, the 14 GCTBs showed the most homogeneous DNA methylation patterns and accordingly grouped together (Fig. 3, heatmap with dendrogram).

We next analyzed copy number data derived from the DNA methylation arrays (Fig. 3, lower panel). *H3F3A* mutant osteosarcomas frequently showed a gain of chromosome arm 8q involving the c-*MYC* locus (3/6) and a distinct deletion of 3q13.31 involving *LSAMP* (2/6). However, c-*MYC* amplification, 6p12–21 amplification or *chromothripsis*, both hallmark copy number alterations in osteosarcomas, were absent in all six *H3F3A* mutant osteosarcomas. GCTBs lacked any copy number alterations except for the single GCTB with a secondary malignant transformation (Additional file 4: Figure S3).

### *H3F3A* G34 mutant osteosarcomas present with a promoter methylation phenotype

**Fig. 4:**
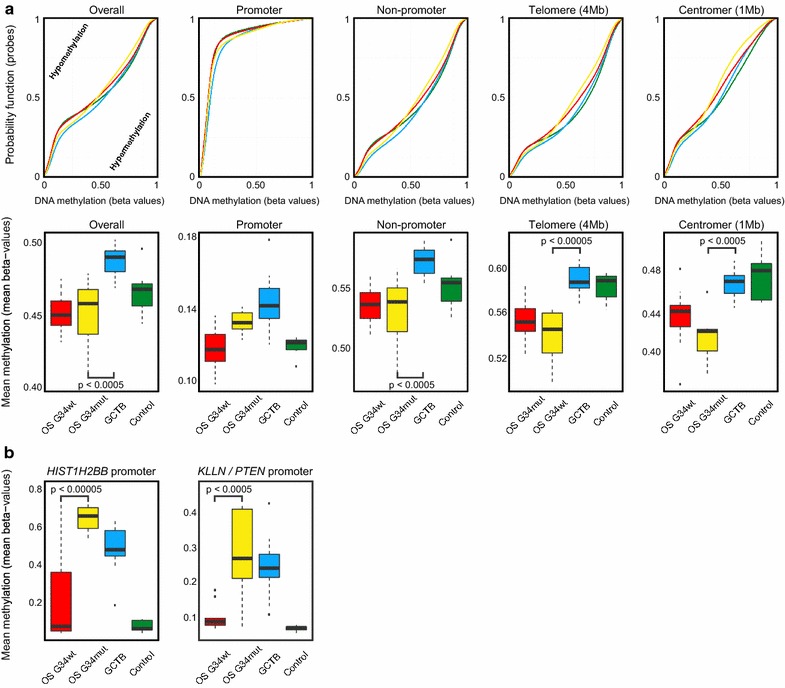
Distinct DNA methylation patterns in osteosarcoma subgroups and giant cell tumors of bone. **a** *Upper part* the empirical cumulative distribution function for DNA methylation levels (beta-values) is plotted individually for each subgroup. *Lower part* DNA methylation levels (mean beta values) regarding different genomic regions of individual osteosarcoma methylation subgroups. **b** Further DNA methylation analyses identified the *HIST1H2BB* and *KLNN*/PTEN promoter as the most differentially methylated region when comparing the *H3F3A* G34 mutant osteosarcoma subgroup (OS G34) with the H3.3 wild-type osteosarcoma group (OS H3.3 wt). Significant difference to the *H3F3A* G34 mutant osteosarcoma subgroup (OS G34) is indicated

Given the distinct DNA methylation patterns of *H3F3A* G34 mutated osteosarcomas we next examined differences in DNA methylation in specific genomic regions (Fig. 4). *H3F3A* G34 mutant and H3.3 wild-type osteosarcomas showed widespread hypomethylation across the whole genome, especially in non-promoter regions, which was particularly prominent at centromeres and telomeres (Fig. 4a). However, *H3F3A* G34 mutant osteosarcomas had higher methylation levels in promoter regions compared with their H3.3 wild-type counterparts. Specifically, we found that the promoter region of the genes *HIST1H2BB* (p < 0.00005) and *KLLN*/*PTEN* (p < 0.0005) showed significantly higher methylation levels in *H3F3A* G34 mutant osteosarcomas (Fig. 4b).

## Discussion

H3.3 mutations in bone tumors occur with an exceedingly high frequency in GCTBs and chondroblastomas [18, 19]. We herein present for the first time a clinical and molecular characterization of six *H3F3A* mutant de novo osteosarcomas and suggest that H3.3 mutations might define a distinct molecular subtype in the heterogeneous group of osteosarcomas.

Looking at the *H3F3A* G34 mutational status and the age distribution alone raises the possibility that the group of H3.3 mutant osteosarcomas may be composed of GCTBs presenting with Denosumab-therapy induced changes mimicking an osteosarcoma-like histology, may have undergone malignant progression or may represent the very rare de novo malignant variant of GCTBs, the latter two of which are known for their occurrence in elderly [23–25]. It is also evident that osteosarcomas in elderly are often of secondary origin, which would further argue in this direction [1].

Indeed, at least for the *H3F3A* G34 mutant osteosarcoma cases presented here, there is no absolute certainty that they might not represent the most undifferentiated end in a spectrum of GCTB differentiation. However, none of the *H3F3A* mutant osteosarcomas presented with distinct radiological and histological features resembling GCTBs or its malignant variant, even though the radiologic appearance of malignant GCTBs may be challenging to distinguish from osteosarcomas [26]. We also cannot preclude with absolute certainty that areas reminiscent for a GCTB were missed due to a sampling bias in the *H3F3A* mutant osteosarcomas, which would argue for the extremely rare de novo malignant variant of GCTBs. However, this can be considered very unlikely. Furthermore, the patients were treated according to standard treatment protocols for osteosarcoma, thereby excluding Denosumab-induced changes. It is also unlikely that a precursor lesion underwent malignant transformation, since medical records were empty in this regard (Table 1). Therefore, taking all information into account, the diagnosis conventional osteosarcoma has been retained in all six H3.3 mutant cases. The *H3F3A* mutant osteosarcoma cases of this study predominantly occurred at higher patient age. This finding might explain why other osteosarcoma studies focusing on pediatric patients alone did not find H3.3 mutations at all [8]. However, larger future studies will be required to confirm that *H3F3A* mutant osteosarcomas are rare or even absent in children and adolescent patients. Interestingly, the age distribution of the H3.3 mutant osteosarcomas is in contrast to H3.3 mutant brain tumors, which typically present at younger age than their wild-type adult counterparts [15, 27]. This observation coupled with evidence from studies about *H3F3A* G34 mutant GCTBs might suggest an entity dependent age distribution of H3.3 mutant tumors [18, 19].

Our integrated approach revealed several molecular alterations in *H3F3A* mutant osteosarcomas that are typical for high-grade osteosarcomas. A homozygous deletion of 3q13.31 carrying the *LSAMP* gene was seen in two of the six *H3F3A* mutant osteosarcomas. One of them additionally carried a *TP53* deletion, a prominent tumor suppressor gene known to be involved in the development of osteosarcomas [8]. *LSAMP* is a tumor suppressor that is frequently inactivated in conventional osteosarcomas and linked to increased proliferation. It is also a predictor for an unfavorable biological behavior [28]. Thus, an aggressive biological behavior typically seen in conventional osteosarcomas might also be assumed for osteosarcomas with *H3F3A* mutations. Accordingly, three of four *H3F3A* mutant osteosarcoma patients with follow-up data available showed an unfavorable clinical course. Two patients died of the disease shortly after diagnosis. One patient presented with widespread metastases and progressive disease (Table 1).

A gain of chromosome 8q bearing the c-*MYC* locus was detected in three of six *H3F3A* mutant osteosarcomas. Amplification of c-*MYC*, a member of the *MYC* transcription factor family tightly involved in the modulation of gene expression and a strong oncogene, has been frequently observed in osteosarcomas [7, 8, 10]. It has also been shown in mice that c-*MYC* overexpression in mesenchymal bone stem cells may induce osteosarcomas [29]. The gain of 8q might confer a tumor propagating gene dosage effect, although c-*MYC* is not amplified in the six *H3F3A* mutant osteosarcomas presented here. In this context, other *MYC* gene family members may also be pivotal in the development of *H3F3A* mutant osteosarcomas. Brain tumors with *H3F3A* G34 mutations have been associated with an increased expression of *MYCN*, another transcription factor well known for its oncogenic capacity [30]. Expression analysis might give further insights whether c-*MYC*, *MYCN*, and/or other oncogenes orchestrate together in *H3F3A* G34 mutant osteosarcomas. The *H3F3A* G34 mutation alone will probably not be sufficient to induce osteosarcomas, since GCTBs carrying such mutations usually do not undergo malignant transformation [1].

We also detected one osteosarcoma with a *H3F3A* K27M mutation. This mutation was initially found in diffuse pontine midline gliomas and has recently been described also in other brain tumor entities [27, 31]. The *H3F3A* K27M mutation has been demonstrated leading to an enzymatic inhibition of EZH2 in brain tumors, a catalytic subunit of Polycomb Repressor Complex 2 [32]. EZH2 catalyzes methylation of histone H3 at lysine 27 and mediates gene silencing of target genes via local chromatin reorganization. Dysregulated EZH2 activity is seen in many cancer types [33]. We here report the first H3 K27M mutant tumor outside the nervous system. It therefore remains to be determined whether the underlying molecular mechanisms of H3.3 K27M mutation described in brain tumors may be adapted to other cancer types.

Interestingly, the DNA methylation analysis revealed several differing aspects in *H3F3A* mutant osteosarcomas compared to their H3.3 wild-type counterparts. Our data indicate that the DNA methylation signature of G34 mutant osteosarcomas is different to their conventional wild-type counterparts, but more closely related to GCTBs. However, this difference was only seen for G34 mutant cases, since the single K27M mutant osteosarcoma of this series was indistinguishable from H3.3 wild-type cases. On the one hand, the close relation between *H3F3A* G34 mutant osteosarcomas and *H3F3A* G34W/L mutant GCTBs might suggest a similar cell of origin. On the other hand, their close relation at the methylation level might reflect similar changes in the epigenetic landscape caused by the *H3F3A* G34 mutation or a responsible higher order mechanism. However, the DNA methylation profile of a dedifferentiated chondrosarcoma, in which we detected a *H3F3A* G34L mutation, was neither related to GCTBs, nor related to *H3F3A* G34W/R mutant osteosarcomas (data not shown).

In-depth analysis of the methylome data revealed widespread DNA hypomethylation in both, *H3F3A* G34 mutant and wild-type osteosarcomas. DNA hypomethylation is generally more pronounced with increased aggressiveness, which might explain this pattern [34]. However, the hypomethylation phenotype was less pronounced within the promoter regions in *H3F3A* G34 mutant osteosarcomas compared to their wild-type counterparts. The most differentially methylated promoter regions involved the *HIST1H2BB* and *KLNN/PTEN* genes. Both showed significantly higher methylation in *H3F3A* G34 mutant osteosarcomas. The role of *HIST1H2BB* in cancer is largely unknown. *HIST1H2BB* is an integral element of the nucleosome complex and hence, impaired expression could disrupt the physiological status and affect nucleosome remodelling [35]. *KLNN* is located juxtaposed to *PTEN* on chromosome 10q23 and both genes share the same promoter site [36]. Accordingly, both genes probably might be affected in *H3F3A* G34 mutant osteosarcomas. An important role of *PTEN* in the development of osteosarcomas has not been described yet, although *PTEN* alteration has been observed [8, 10, 37]. The neighboring gene *KLLN* functions at the pericentric region of chromosomes. *KLLN* organizes the heterochromatin by maintaining the H3K9 trimethylation and thereby contributes to chromosomal stability. Loss of *KLLN* resulted in the dysregulation of pericentric heterochromatin with consequent chromosomal instability and numerical chromosomal aberrations in vitro [38]. Interestingly, hypomethylation was significantly pronounced around the pericentric region in *H3F3A* G34 mutant osteosarcomas compared with GCTBs. Whether the hypomethylation phenotype may be a surrogate for pericentric instability and the role of *KLLN* in context of H3.3 mutations will be subject of future projects.

## Conclusions

Taken together, our data confirm that detection of *H3F3A* mutations in bone tumors does not exclude malignancy in individual cases, especially in older patients. *H3F3A* mutant conventional high-grade osteosarcomas may radiologically, histologically and karyotypically be indistinguishable from conventional high-grade osteosarcomas, but present with distinct epigenetic features.

## Additional files

**Additional file 1: Table S1.** Clinical information of the entire study cohort.

**Additional file 2: Figure S1.** Radiological data of *H3F3A* mutant osteosarcomas. (A) Case 77896: anteroposterior and lateral radiograph of the left wrist. Arrows indicate toward the lesion with permeated growth pattern (moth-eaten) and periosteal reaction. (B) Case 84676: anteroposterior and lateral radiograph (upper panel) with arrows indicating a large soft tissue mass with prominent mineralization and corresponding sagittal T1- and fat-saturated proton-weighted MR image (lower panel) of the right knee. The tumor involves the adjacent epiphyses through the epiphyseal growth plate (arrow). (C) Case 94316: anteroposterior and lateral radiograph (upper panel) and corresponding sagittal T1-weighted MR image (lower panel left) of the right knee. The MR image shows the intact bone cortex in the cranial part of the lesion (white arrows), but cortical disruption with “wrap-around” sign in the caudal part of the lesion. Anteroposterior radiograph (lower panel right) shows the local recurrence in the soft tissue adjacent to the knee joint prosthesis. (D) Case 84712: anteroposterior and lateral radiograph (upper panel) and corresponding axial T1-weighted and sagittal fat-saturated proton-weighted MR image (lower panel) of the right knee. Arrows indicate periosteal reaction and cortical disruption. (E) Case 94314: anteroposterior radiograph (upper panel, left), coronal fat-saturated T1-weighted MR image after administration of gadolinium contrast agent (upper panel, right) and coronal T1-weighted MR image (lower panel, left) of the right hip. Thickened bone cortex (arrow-heads, hypointense fibrous structure) is wrapped by the lesion. Anteroposterior radiograph (lower panel, right) after hip joint replacement.

**Additional file 3: Figure S2.** Histological data of the six *H3F3A* mutant osteosarcomas (A–F) and the single giant cell tumor of bone with malignant transformation (G).

**Additional file 4: Figure S3.** Copy number plots of the six *H3F3A* mutant osteosarcomas (A–F), one prototypic H3.3 wild-type osteosarcoma with an scattered chromosome arm 2q and the entire chromosome 3 indicating chromothripsis (G), one prototypical giant cell tumor of bone with a flat profile, (H) and one malignant giant cell tumor of bone with hints for a segmental loss of chromosome arm 2q and segmental gain of 7q (I). Abbreviations: OS = osteosarcoma; GCTB = giant cell tumor of bone.

## Abbreviations

OS: osteosarcoma
GCTB: giant cell tumor of bone
H3.3: histone 3.3
ALT: alternative lengthening of telomeres
ATRX: α-thalassaemia/mental retardation syndrome X-linked
DAXX: death-domain associated protein
H3F3A/B: histone 3 family 3 A/B
DKFZ: german cancer research center
SD: standard deviation
MR: magnetic resonance
