# Supplementary material for: Histone 3.3 hotspot mutations in conventional osteosarcomas: a comprehensive clinical and molecular characterization of six H3F3A mutated cases
Source: Clin Sarcoma Res. 2017 May 4;7:9. doi: 10.1186/s13569-017-0075-5 (PMC5418758; doi:10.1186/s13569-017-0075-5)
Supplement: Supplementary file 1 — Additional file 1: Table S1. Clinical information of the entire study cohort. [file 13569_2017_75_MOESM1_ESM.pdf]

Supplementary Table S1

ID	AGE	SEX	MANIFESTATION	450k	TUMOR-TYPE	Localization	H3F3A	H3F3B
58166	25	f	Metastasis	no	Osteosarcoma	Head	wt	wt
67148	17	f	Metastasis	no	Osteosarcoma	Lung	wt	wt
67514	15	f	Metastasis	no	Osteosarcoma	Lung	wt	wt
67760	16	f	Metastasis	no	Osteosarcoma	Lung	wt	wt
70708	17	m	Metastasis	no	Osteosarcoma	Scapular	wt	wt
77854	13	f	Primary	no	Osteosarcoma	Extremity (upper)	wt	wt
77856	6	f	Primary	yes	Osteosarcoma	Extremity (lower)	wt	wt
77858	18	m	Primary	yes	Osteosarcoma	Extremity (upper)	wt	wt
77860	9	f	Primary	yes	Osteosarcoma	Extremity (lower)	wt	wt
77862	15	f	Primary	no	Osteosarcoma	Extremity (lower)	wt	wt
77864	16	m	Primary	yes	Osteosarcoma	Extremity (upper)	wt	wt
77866	16	m	Primary	yes	Osteosarcoma	Extremity (lower)	wt	wt
77868	18	m	Primary	yes	Osteosarcoma	Extremity (lower)	wt	wt
77870	14	m	Primary	yes	Osteosarcoma	Extremity (lower)	wt	wt
77872	12	m	Primary	yes	Osteosarcoma	Extremity (lower)	wt	wt
77894	20	m	Primary	yes	Osteosarcoma	Extremity (lower)	wt	wt
77896	59	m	Primary	yes	Osteosarcoma	Extremity (upper)	G34W homozygous	wt
77900	29	m	Primary	yes	Osteosarcoma	Extremity (lower)	wt	wt
77902	56	f	Primary	no	Osteosarcoma	Extremity (lower)	wt	wt
77906	59	f	Primary	no	Osteosarcoma	Extremity (lower)	wt	wt
77908	34	f	Primary	yes	Osteosarcoma	Extremity (lower)	wt	wt
78510	17	f	Metastasis	no	Osteosarcoma	Lung	wt	wt
79338	2	f	Metastasis	no	Osteosarcoma	Lung	wt	wt
79354	15	f	Primary	yes	Osteosarcoma	Extremity (lower)	wt	wt
79414	15	m	Primary	yes	Osteosarcoma	NA	wt	wt
79416	14	f	Primary	yes	Osteosarcoma	Scapula	wt	wt
79420	65	m	Primary	yes	Osteosarcoma	Spine	wt	wt
79428	75	m	Primary	yes	Osteosarcoma	Extremity (lower)	G34W hemizygous	wt
79490	9	m	Primary	yes	Osteosarcoma	Extremity (lower)	wt	wt
79502	35	m	Primary	yes	Osteosarcoma	Extremity (upper)	wt	wt
79506	12	f	Primary	yes	Osteosarcoma	Extremity (lower)	wt	wt
79602	24	f	Primary	yes	Osteosarcoma	Extremity (lower)	wt	wt
79612	12	f	Primary	yes	Osteosarcoma	Extremity (lower)	wt	wt
79680	78	m	Primary	yes	Osteosarcoma	Pelvis	wt	wt
79922	72	f	Metastasis	no	Osteosarcoma	Head	wt	wt
80844	27	m	Recurrence	yes	Osteosarcoma	Head	wt	wt
81968	27	f	Primary	yes	Giant cell tumor of bone	Extremity (upper)	G34W heterozygous	wt

81996	43	m	Primary	no	Osteosarcoma	Extremity (lower)	wt	wt
82002	44	m	Primary	no	Osteosarcoma	Extremity (lower)	wt	wt
82004	67	f	Metastasis	no	Osteosarcoma	Extremity (lower)	wt	wt
82006	75	f	Primary	no	Osteosarcoma	Pelvis	wt	wt
82050	45	f	Recurrence	no	Osteosarcoma	Extremity (lower)	wt	wt
82052	14	f	Primary	yes	Osteosarcoma	Extremity (lower)	wt	wt
82054	15	m	Primary	no	Osteosarcoma	Extremity (upper)	wt	wt
82056	19	m	Primary	no	Osteosarcoma	Extremity (lower)	wt	wt
82058	77	f	Primary	no	Osteosarcoma	Extremity (lower)	wt	wt
82062	39	m	Primary	no	Osteosarcoma	Extremity (lower)	wt	wt
82070	21	m	Primary	yes	Osteosarcoma	Extremity (lower)	wt	wt
82072	73	m	Primary	no	Osteosarcoma	Extremity (lower)	wt	wt
82074	16	m	Primary	yes	Osteosarcoma	Extremity (lower)	wt	wt
82078	58	m	Primary	no	Osteosarcoma	Extremity (lower)	wt	wt
82080	42	m	Primary	no	Osteosarcoma	Extremity (lower)	wt	wt
82154	86	m	Primary	no	Osteosarcoma	Thorax	wt	wt
82160	68	f	Primary	no	Osteosarcoma	Extremity (lower)	wt	wt
82162	62	f	Primary	no	Osteosarcoma	Extremity (lower)	wt	wt
82170	57	m	Primary	yes	Osteosarcoma	Extremity (lower)	wt	wt
82174	61	f	Primary	no	Osteosarcoma	Pelvis	wt	wt
82298	33	m	Primary	yes	Giant cell tumor of bone	Extremity (lower)	G34W heterozygous	wt
82300	29	m	Primary	yes	Giant cell tumor of bone	Extremity (upper)	G34W heterozygous	wt
82340	75	f	Primary	yes	Giant cell tumor of bone	Extremity (lower)	G34W heterozygous	wt
82344	32	m	Primary	yes	Giant cell tumor of bone	Extremity (upper)	G34W heterozygous	wt
82346	18	m	Recurrence	yes	Giant cell tumor of bone (malignant)	Extremity (upper)	G34W heterozygous	wt
82348	20	f	Primary	yes	Giant cell tumor of bone	Pelvis	G34W heterozygous	wt
82350	27	f	Primary	yes	Giant cell tumor of bone	Extremity (upper)	G34W heterozygous	wt
82498	23	f	Metastasis	no	Osteosarcoma	Gluteal	wt	wt
83166	34	f	Primary	yes	Giant cell tumor of bone	Extremity (lower)	G34W heterozygous	wt
83168	58	m	Primary	yes	Giant cell tumor of bone	Extremity (lower)	G34W heterozygous	wt
83170	59	m	Primary	yes	Giant cell tumor of bone	Extremity (upper)	G34W heterozygous	wt
83244	26	f	Primary	yes	Giant cell tumor of bone	Extremity (upper)	G34W heterozygous	wt
84028	22	f	Primary	yes	Giant cell tumor of bone	Extremity (lower)	G34W heterozygous	wt
84674	38	m	Primary	no	Osteosarcoma	Extremity (lower)	wt	wt
84676	71	m	Primary	yes	Osteosarcoma	Extremity (lower)	G34W heterozygous	wt
84678	9	m	Primary	no	Osteosarcoma	Extremity (lower)	wt	wt
84680	13	m	Primary	no	Osteosarcoma	Extremity (upper)	wt	wt
84682	19	m	Primary	no	Osteosarcoma	Extremity (lower)	wt	wt
84684	31	f	Primary	no	Osteosarcoma	Extremity (lower)	wt	wt

84686	5	f	Primary	no	Osteosarcoma	Extremity (lower)	wt	wt
84688	10	m	Primary	no	Osteosarcoma	Extremity (lower)	wt	wt
84690	14	m	Primary	no	Osteosarcoma	Extremity (lower)	wt	wt
84692	36	f	Primary	no	Osteosarcoma	Extremity (lower)	wt	wt
84694	13	m	Primary	no	Osteosarcoma	Extremity (lower)	wt	wt
84696	83	m	Primary	no	Osteosarcoma	Extremity (lower)	wt	wt
84698	14	m	Primary	no	Osteosarcoma	Extremity (lower)	wt	wt
84700	14	f	Primary	no	Osteosarcoma	Extremity (lower)	wt	wt
84702	7	m	Primary	no	Osteosarcoma	Extremity (upper)	wt	wt
84704	13	f	Primary	no	Osteosarcoma	Extremity (lower)	wt	wt
84706	42	m	Primary	no	Osteosarcoma	Pelvis	wt	wt
84708	11	m	Primary	no	Osteosarcoma	NA	wt	wt
84710	11	f	Primary	no	Osteosarcoma	Extremity (lower)	wt	wt
84712	34	f	Recurrence	yes	Osteosarcoma	Extremity (lower)	G34W heterozygous	wt
84714	47	m	Primary	no	Osteosarcoma	Extremity (upper)	wt	wt
85180	29	f	Primary	yes	Giant cell tumor of bone	Extremity (lower)	G34L heterozygous	wt
85484	20	f	Primary	yes	Osteosarcoma	Jaw	wt	wt
85550	16	m	Metastasis	no	Osteosarcoma	Lung	wt	wt
85914	32	m	Metastasis	no	Osteosarcoma	Trunk	wt	wt
85922	30	m	Primary	yes	Osteosarcoma	Spine	wt	wt
86710	18	f	Metastasis	no	Osteosarcoma	Lung	wt	wt
90676	4	m	Metastasis	no	Osteosarcoma	Lung	wt	wt
91092	23	m	Metastasis	no	Osteosarcoma	Thorax	wt	wt
91264	15	f	Metastasis	no	Osteosarcoma	Lung	wt	wt
91384	19	m	Metastasis	no	Osteosarcoma	Lung	wt	wt
94296	12	m	Primary	no	Osteosarcoma	Extremity (lower)	wt	wt
94298	20	m	Primary	no	Osteosarcoma	Extremity (lower)	wt	wt
94300	12	m	Primary	no	Osteosarcoma	Extremity (upper)	wt	wt
94310	75	f	Primary	no	Osteosarcoma	Extremity (upper)	wt	wt
94312	48	f	Primary	no	Osteosarcoma	Extremity (lower)	wt	wt
94314	34	f	Primary	yes	Osteosarcoma	Extremity (lower)	K27M heterozygous	wt
94316	75	m	Primary	yes	Osteosarcoma	Extremity (upper)	G34R heterozygous	wt
94318	5	f	Primary	no	Osteosarcoma	Extremity (lower)	wt	wt
94320	34	m	Primary	no	Osteosarcoma	Extremity (lower)	wt	wt
94324	15	m	Primary	no	Osteosarcoma	Extremity (lower)	wt	wt
94326	15	f	Primary	no	Osteosarcoma	Extremity (lower)	wt	wt
94538	13	m	Primary	no	Osteosarcoma	Head	wt	wt
94542	7	m	Primary	no	Osteosarcoma	Extremity (lower)	wt	wt
94544	14	f	Primary	no	Osteosarcoma	Extremity (lower)	wt	wt

94566	14	m	Primary	no	Osteosarcoma	Extremity (lower)	wt	wt
94572	9	f	Primary	no	Osteosarcoma	Extremity (lower)	wt	wt
94574	13	m	Primary	no	Osteosarcoma	Extremity (upper)	wt	wt
94576	69	f	Primary	no	Osteosarcoma	Extremity (upper)	wt	wt
94578	76	m	Primary	no	Osteosarcoma	Jaw	wt	wt
