# Supplementary figures and images for: Histone 3.3 hotspot mutations in conventional osteosarcomas: a comprehensive clinical and molecular characterization of six H3F3A mutated cases
Source: Clin Sarcoma Res. 2017 May 4;7:9. doi: 10.1186/s13569-017-0075-5 (PMC5418758; doi:10.1186/s13569-017-0075-5)

Case 77896 (A)

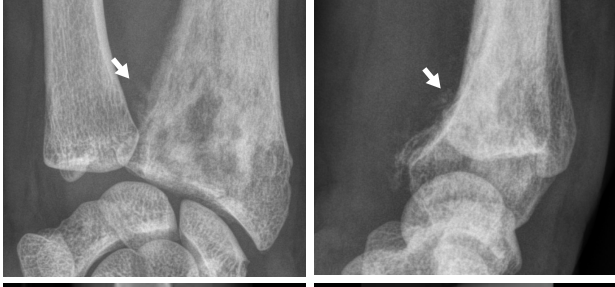

Case 94316 (C)

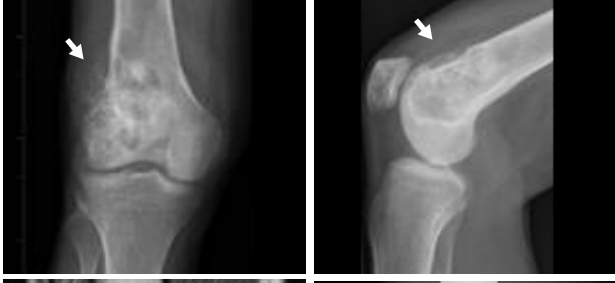

Case 94314 (E)

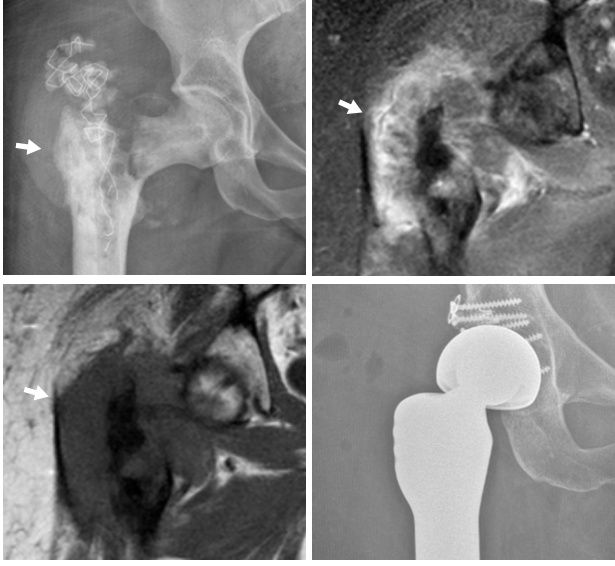

Case 84676 (B)

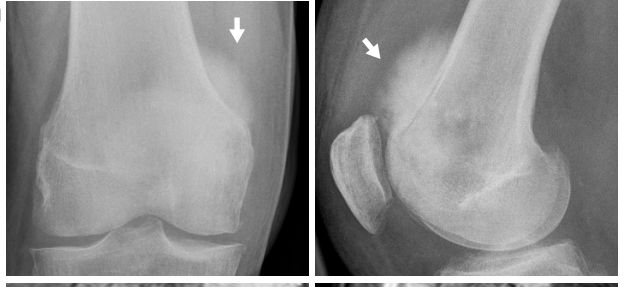

Case 84712 (D)

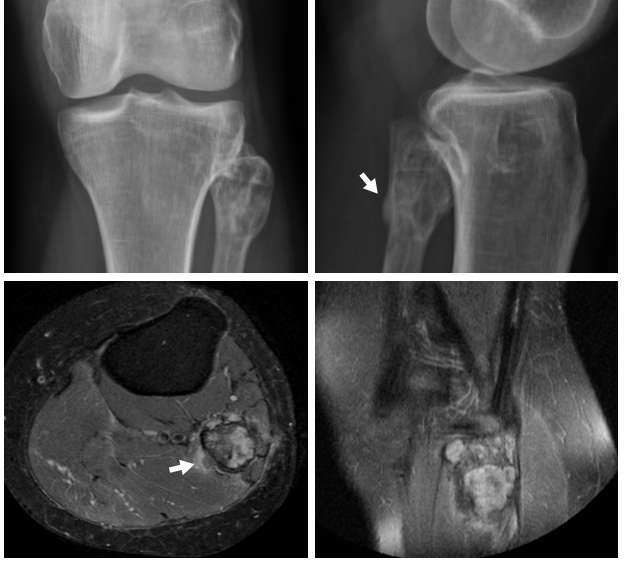

Supplement: Supplementary file 2 — Additional file 2: Figure S1. Radiological data of H3F3A mutant osteosarcomas. (A) Case 77896: anteroposterior and lateral radiograph of the left wrist. Arrows indicate toward the lesion with permeated growth pattern (moth-eaten) and periosteal reaction. (B) Case 84676: anteroposterior and lateral radiograph (upper panel) with arrows indicating a large soft tissue mass with prominent mineralization and corresponding sagittal T1- and fat-saturated proton-weighted MR image (lower panel) of the right knee. The tumor involves the adjacent epiphyses through the epiphyseal growth plate (arrow). (C) Case 94316: anteroposterior and lateral radiograph (upper panel) and corresponding sagittal T1-weighted MR image (lower panel left) of the right knee. The MR image shows the intact bone cortex in the cranial part of the lesion (white arrows), but cortical disruption with “wrap-around” sign in the caudal part of the lesion. Anteroposterior radiograph (lower panel right) shows the local recurrence in the soft tissue adjacent to the knee joint prosthesis. (D) Case 84712: anteroposterior and lateral radiograph (upper panel) and corresponding axial T1-weighted and sagittal fat-saturated proton-weighted MR image (lower panel) of the right knee. Arrows indicate periosteal reaction and cortical disruption. (E) Case 94314: anteroposterior radiograph (upper panel, left), coronal fat-saturated T1-weighted MR image after administration of gadolinium contrast agent (upper panel, right) and coronal T1-weighted MR image (lower panel, left) of the right hip. Thickened bone cortex (arrow-heads, hypointense fibrous structure) is wrapped by the lesion. Anteroposterior radiograph (lower panel, right) after hip joint replacement. [file 13569_2017_75_MOESM2_ESM.pdf]

Case 82346

Case 79428

Case 94314

Case 84712

Case 94316

Case 84676

Case 77896

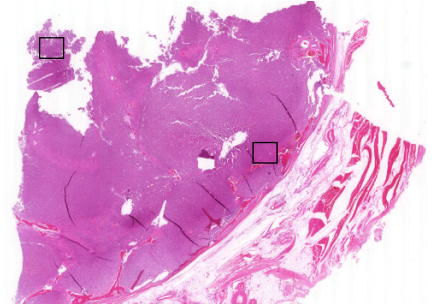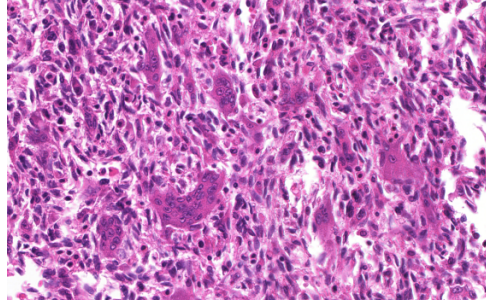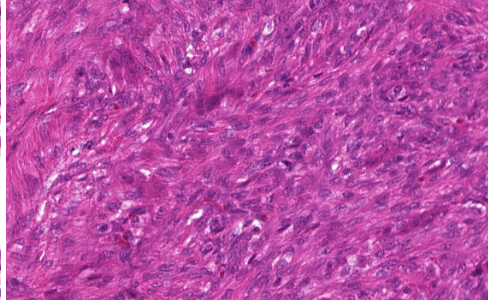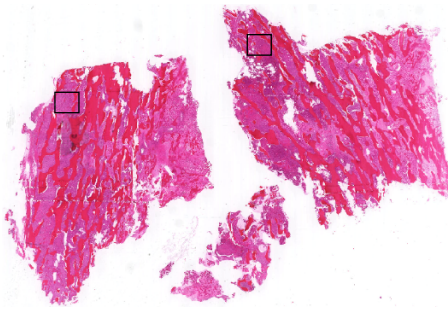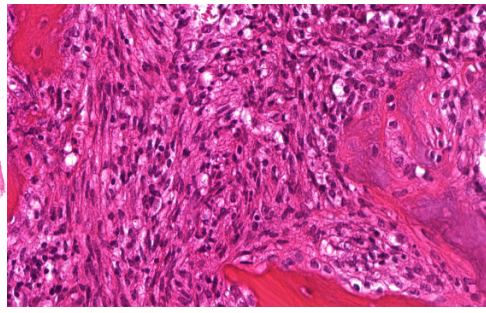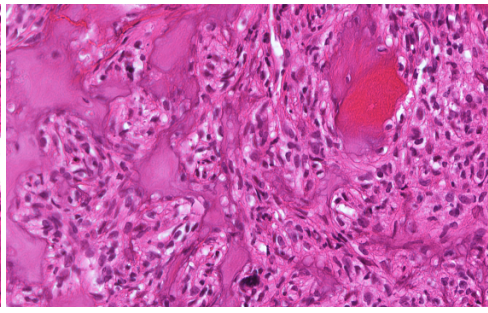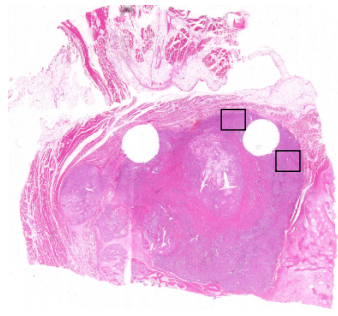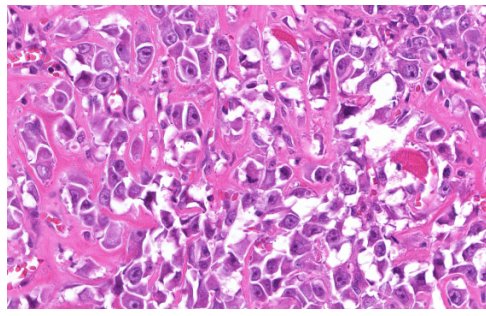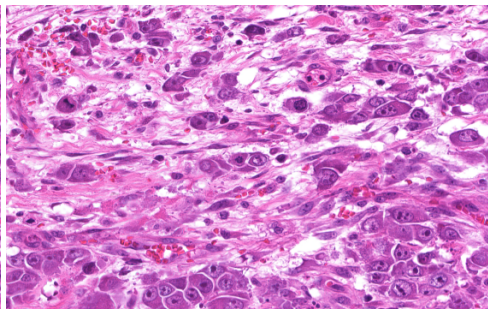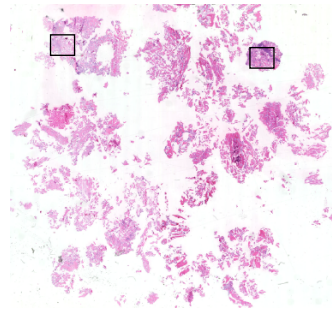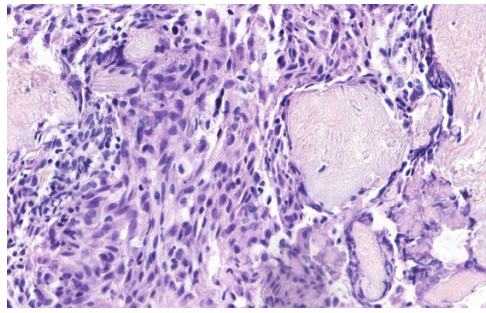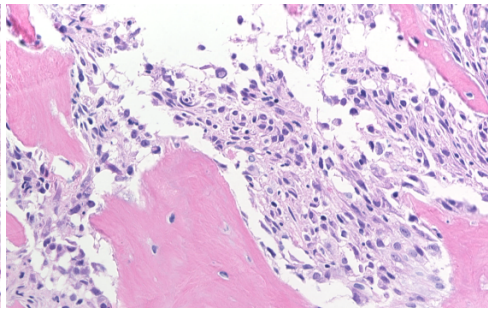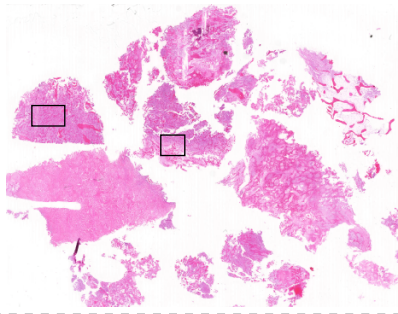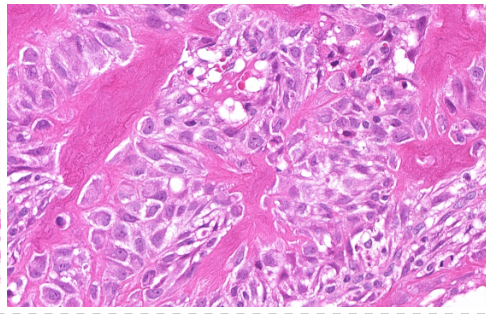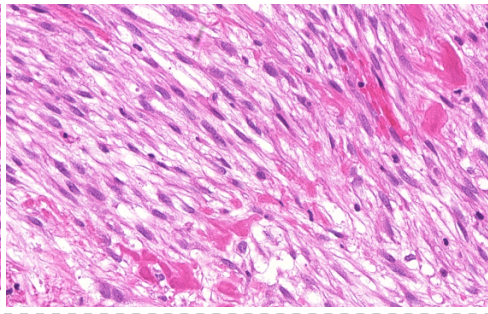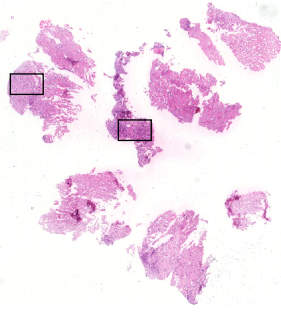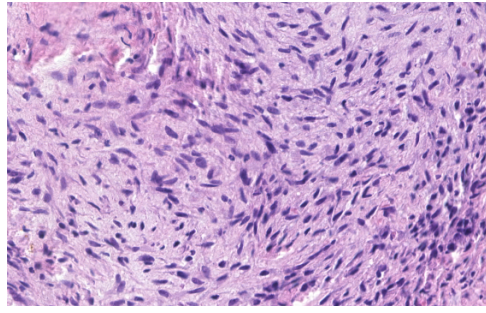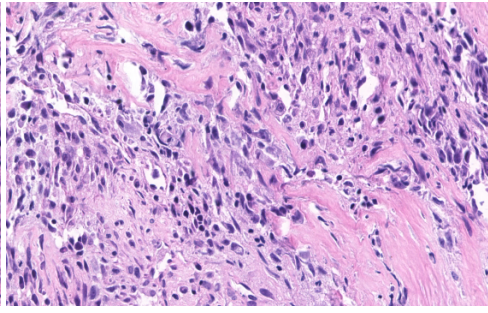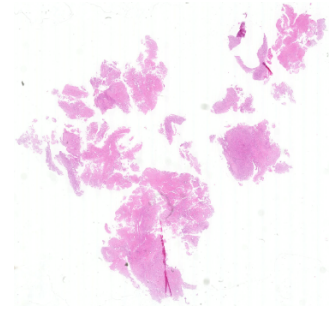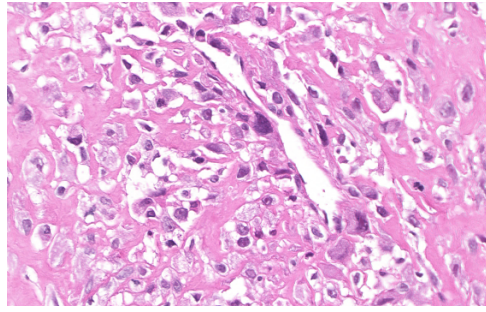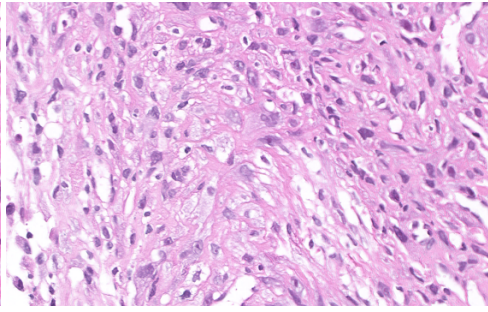

Supplement: Supplementary file 3 — Additional file 3: Figure S2. Histological data of the six H3F3A mutant osteosarcomas (A–F) and the single giant cell tumor of bone with malignant transformation (G). [file 13569_2017_75_MOESM3_ESM.pdf]
