# Supplementary material for: Histone 3.3 hotspot mutations in conventional osteosarcomas: a comprehensive clinical and molecular characterization of six H3F3A mutated cases
Source: Clin Sarcoma Res. 2017 May 4;7:9. doi: 10.1186/s13569-017-0075-5 (PMC5418758; doi:10.1186/s13569-017-0075-5)
Supplement: Supplementary file 4 — Additional file 4: Figure S3. Copy number plots of the six H3F3A mutant osteosarcomas (A–F), one prototypic H3.3 wild-type osteosarcoma with an scattered chromosome arm 2q and the entire chromosome 3 indicating chromothripsis (G), one prototypical giant cell tumor of bone with a flat profile, (H) and one malignant giant cell tumor of bone with hints for a segmental loss of chromosome arm 2q and segmental gain of 7q (I). Abbreviations: OS = osteosarcoma; GCTB = giant cell tumor of bone. [file 13569_2017_75_MOESM4_ESM.pdf]

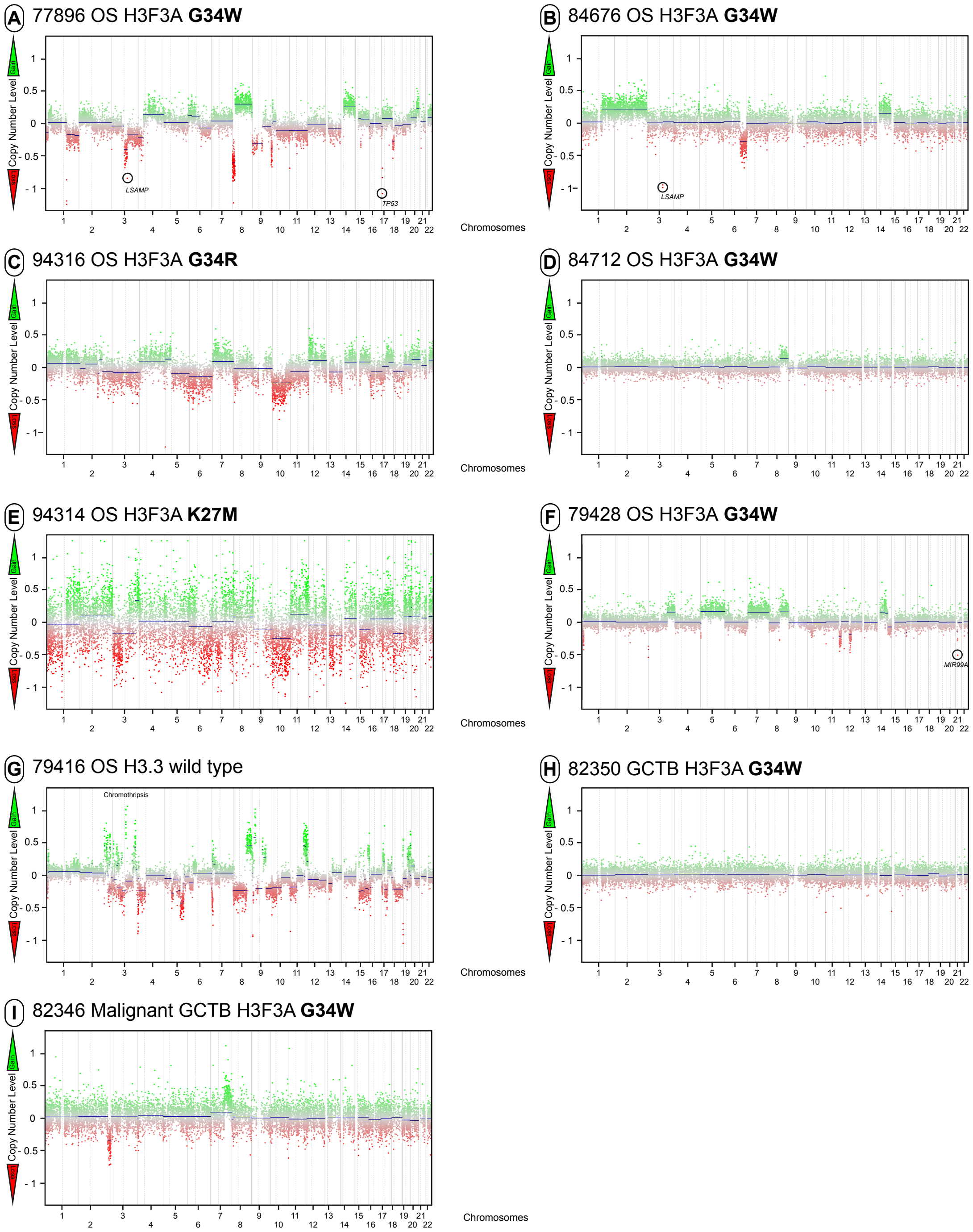
A 77896 OS H3F3A G34W
B 84676 OS H3F3A G34W
C 94316 OS H3F3A G34R
D 84712 OS H3F3A G34W
E 94314 OS H3F3A K27M
F 79428 OS H3F3A G34W
G 79416 OS H3.3 wild type
H 82350 GCTB H3F3A G34W
I 82346 Malignant GCTB H3F3A G34W
Copy Number Level
Chromosomes
LSAMP
TP53
MIR99A
Chromothripsis
